# Visualized, radiation-free diagnosis and treatment of biliary stone complexes from migrated surgical clips via peroral cholangioscopy

**DOI:** 10.1055/a-2541-2012

**Published:** 2025-03-03

**Authors:** Yu Tang, Junyi Zhuo, Min Lin, Xianfei Zhong

**Affiliations:** 1 Department of Gastroenterology, People’s Hospital of Leshan, Southwest Medical University, Leshan, China

Postoperative clip migration into the common bile duct (CBD) is a rare complication of laparoscopic biliary surgery. The migrated clips may serve as niduses for stone formation 1 . This is a report of cholangioscopy-guided diagnosis and treatment of biliary stone-clip complexes.

A 41-year-old man was referred to our hospital with bile duct stones. Two years earlier he had undergone laparoscopic bile duct exploration and cholecystectomy after a failed endoscopic retrograde cholangiopancreatography (ERCP) aimed at treating large CBD stones. Magnetic resonance cholangiopancreatography (MRCP) revealed a dilated CBD with multiple filling defects, while endoscopic ultrasound (EUS) did not show the typical acoustic shadowing associated with stones ( Fig. 1 ). Peroral cholangioscopy (POC) was performed. Radiation-free biliary cannulation through the non-naive papilla was easily achieved and confirmed by aspiration of bile 2 . Following sphincterotomy, a 9-Fr digital cholangioscope (eyeMax; Microtech, Nanjing, China) was inserted, revealing multiple columnar stones within the distorted CBD lumen ( Fig. 2 ). As the stones were crushed and peeled off by POC-guided electrohydraulic lithotripsy, plastic inner cores were exposed and identified as surgical clips. Consequently, the diagnosis of stone formation around migrated clips was established. Using a through-the-cholangioscope snare, all clips were successfully retrieved. Subsequently, the stones were removed with a wire-guided basket (FG-v432p; Olympus, Tokyo, Japan). Stone clearance was confirmed through POC visualization ( Video 1 ). The patient recovered without any discomfort.

**Fig. 1:**
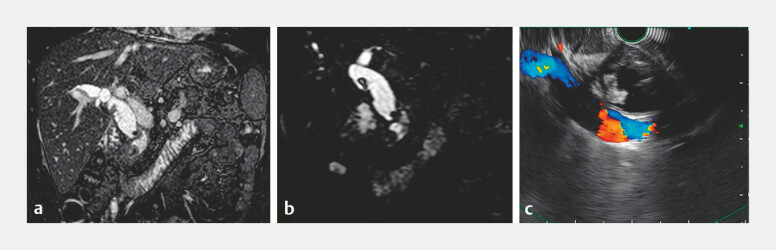
Pre-procedural imaging studies. **a, b** Magnetic resonance cholangiopancreatography showed the dilated and distorted common bile duct with multiple filling defects. **c** Endoscopic ultrasound showed the hyperechoic stone without acoustic shadowing.

**Fig. 2:**
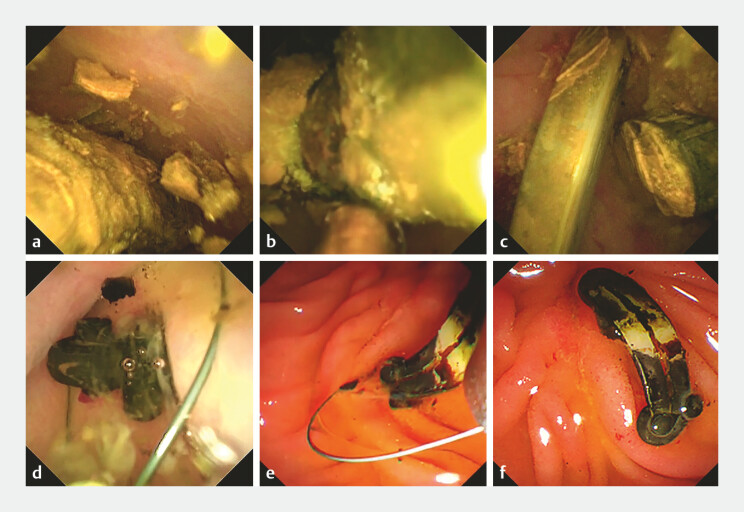
Visualized, radiation-free management of a stone–clip complex using peroral digital cholangioscopy (POC). **a** POC view of a columnar stone. **b** POC-guided electrohydraulic lithotripsy. **c** Adequately exposed clips after thorough fragmentation of the outer stone shell. **d** Grasp of the clip using a dedicated through-the-cholangioscope snare. **e, f** Duodenoscopic view of the removal of a clip.

Visualized, radiation-free diagnosis and treatment of biliary stone complexes from migrated surgical clips via peroral cholangioscopy.Video 1

Stone–clip complexes undetectable on preprocedural imaging studies are also unlikely to be identified during conventional ERCP, increasing the potential risk of impaction during retrieval. POC allows for direct visualization of the biliary system. POC-directed lithotripsy was instrumental in establishing the diagnosis, while thorough fragmentation of the outer stone shell eased the removal of the clips. In conclusion, visualized management of stone–clip complexes utilizing POC is a straightforward, safe, and efficient approach that can be performed without the use of radiation 2 .

Endoscopy_UCTN_Code_TTT_1AR_2AH

## Notes

Endoscopy E-Videos https://eref.thieme.de/e-videos
*E-Videos* is an open access online section of the journal *Endoscopy* , reporting on interesting cases and new techniques in gastroenterological endoscopy. All papers include a high-quality video and are published with a Creative Commons CC-BY license. Endoscopy E-Videos qualify for HINARI discounts and waivers and eligibility is automatically checked during the submission process. We grant 100% waivers to articles whose corresponding authors are based in Group A countries and 50% waivers to those who are based in Group B countries as classified by Research4Life (see: https://www.research4life.org/access/eligibility/ ). This section has its own submission website at https://mc.manuscriptcentral.com/e-videos .
